# Failure of femoral internal fixation device secondary to phosphaturic mesenchymal tumor: a case report and literature review

**DOI:** 10.1186/s12891-025-09066-0

**Published:** 2025-09-01

**Authors:** Ningjing Zeng, Linzhu Zhang, Lian Zhang, Ningzhi Yu, Chuyu Huang, Peng Yang, Yang Lv, Xing Li, Hongliang Liu, Da Guo, Shuchai Xu, Yan Liu

**Affiliations:** 1 The Second Affiliated Hospital of Guangzhou University of Chinese Medicine, Guangzhou, Guangdong China; 2 Shenzhen Children’s Hospital, Shenzhen, Guangdong China; 3 The Second Clinical College of Guangzhou University of Chinese Medicine, Guangzhou, Guangdong China; 4 Guangdong Provincial Second Hospital of Traditional Chinese Medicine, Guangzhou, Guangdong China

**Keywords:** Phosphaturic mesenchymal tumor, Tumor-induced osteomalacia, Femoral fracture cut-and-replace internal fixation, Failure of the femoral internal fixation device, Case report.

## Abstract

**Background:**

Tumor-induced osteomalacia (TIO) is a rare paraneoplastic syndrome caused by mesenchymal tumors that secrete fibroblast growth factor 23 (FGF23). Phosphaturic mesenchymal tumor (PMT) is a novel histopathologic entity that has been identified as a separate cause of TIO. Clinically, PMT is typically diagnosed late due to its rarity.

**Case presentation:**

The paper reports on a patient with a sustained left-sided sub-trochanteric femur fracture in 2018 who subsequently underwent fracture reduction and internal fixation placement. Six years postoperatively, the patient developed a recurrent femoral stem fracture accompanied by internal fixation device failure on the left side of the femur with no apparent causative factors. Based on the patient’s history of multiple fractures, a series of tests were performed and he was diagnosed with a rare disease recognized as TIO. A PET-CT scan conducted prior to surgery revealed that the lesion was located in the right suprapatellar bursa, so the mass was palpated on the body surface and surgically excised.

**Conclusions:**

It is the first reported case of TIO in a patient with internal fixation device failure. Following a thorough review of the patient’s medical history, examination findings, and associated literature, the study concluded that the patient’s long-term osteomalacia and hypophosphatemia would significantly compromise the stability of the internal fixation. Specifically, we determined that the primary tumor lesion in this case was the primary cause of internal fixation failure. Therefore, patients found to have difficult-to-correct hypophosphatemia with multiple fractures in clinical practice should be further investigated for the primary etiology, such as TIO as mentioned in this case, before fracture surgery is performed. Enhancing 68 Ga-DOTA-TATE Positron Emission Tomography/ Computed Tomography (PET/CT) is also recommended to provide a more precise diagnosis of the tumor. For definite tumor lesions, total excisional surgery is considered to be a viable treatment for TIO.

## Background

TIO is a rare paraneoplastic syndrome caused by a mesenchymal tumour that secretes FGF23. The patients present with progressive bone pain, muscle weakness, and fragility fractures. The disease of TIO is characterized by hypophosphatemia, excessive renal phosphate excretion, and abnormal levels of 1,25 dihydroxyvitamin D (1,25(OH)_2_D) [1]. Phosphatidyluremic mesenchymal tumor (PMT) is one of the main tumor types that cause TIO. According to the 2013 edition of the World Health Organization (WHO) classification of soft tissue tumors, PMT is defined as ‘a distinctive mesenchymal tumor usually associated with osteochondrosis, characterized by uniformly colored spindle and stellate cells with small nuclei and indistinct nucleoli, which are richly vascularized, with thick-walled malformations, and can be manifested by calcification and metaplasia’. Almost all cases of TIO are associated with PMT, and PMT tumor cells are able to abnormally secrete large amounts of FGF23, which acts as a key pathogenic factor leading to TIO [2]. PMT, however, is rarely diagnosed for many years due to its rarity and non-specific symptoms. The majority of phosphointerstitial tumors are small in size and can be found nearly anywhere in the body [3]. There is no recognized follow-up and evaluation index for TIO. Besides, there are a limited number of publications available regarding PMT-induced fractures following the placement of an internal fixation. This paper reports a case of PMT after internal fixation placement of femoral fracture combined with secondary fracture due to internal fixation device failure. By reviewing the patient’s test results over the years, we explored the changes in bone metabolism and imaging in patients with PMT, intending to deepen the clinical understanding of the surgical treatment of PMT fractures in the future.

## Case presentation

**Fig. 1:**
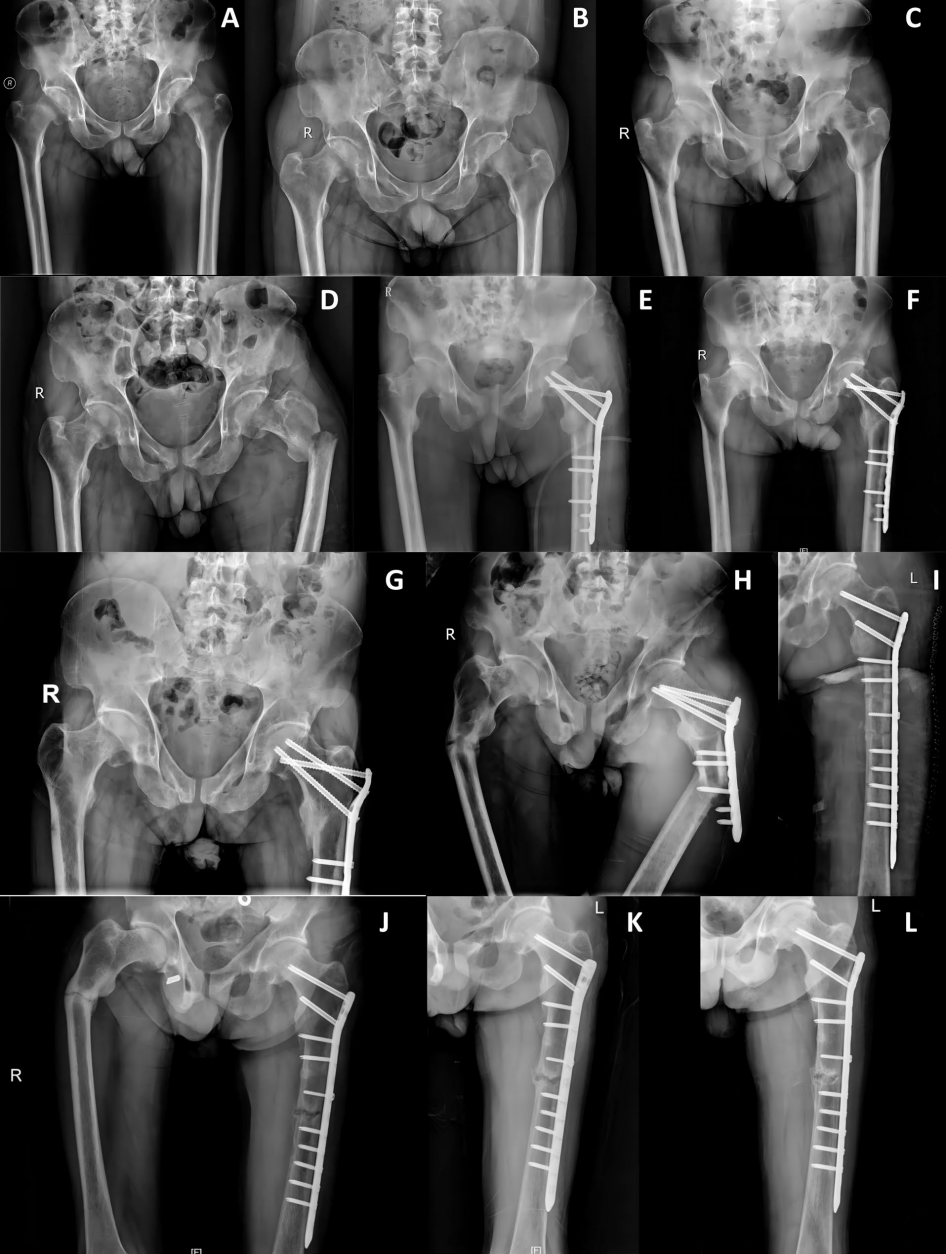
X-rays of the pelvis at different times. **A** Bilateral fractures of the femoral neck occurred in June 2015. **B** The previous fracture has not healed and is more displaced than before in September 2016. **C** The fracture is slightly displaced relative to B and remains unhealed in September 2017. **D** The fracture of the neck of the femur did not fully heal and in March 2018, and a left subrotunda fracture of the femur occurred. **E** Bilateral femoral neck fractures showed healing after post-op in March 2018. **F** The left intertrochanteric fracture of the femur gradually healed, but the plate on the femoral side showed signs of loosening in June 2019. **G** The subtrochanteric fracture has healed, but the right side of the proximal femur showed signs of fracture on the lateral cortex in September 2021. **H** Bilateral fractures of the proximal femur. Notably, the original subtrochanteric fracture on the left side has healed and the plate has loosened, resulting in the development of a new fracture at the femoral stem in March 2024. **I** The left proximal femur fracture with good alignment of the fracture ends after internal fixation with an incised reduction plate was given in March 2024. **J**, **K**, **L** The fractured end of the left femoral stem gradually began to form a bone scab in May, June, and August 2024

**Fig. 2:**
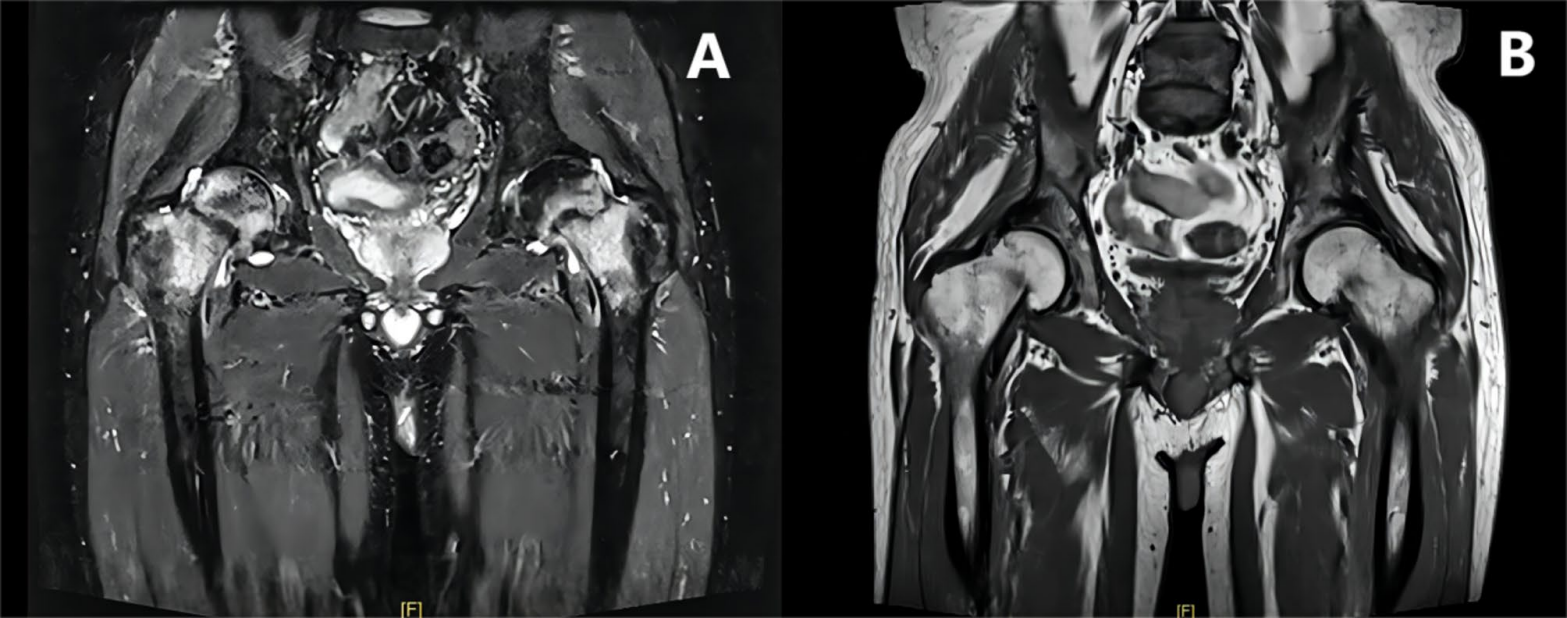
MR images of the patient’s hip joints. **A** Bilateral fractures of the femoral neck occurred in June 2015. **B** Most of the bilateral femoral neck fractures healed in September 2017

The patient in this case, a 60-year-old man, has experienced bilateral hip pain and discomfort since 2015. During 2015–2017, the patient was hospitalized in orthopedics and neurology several times due to pain and discomfort in bilateral hip joints and progressive weakness in both lower limbs. Several imaging examinations suggested bilateral femoral neck fracture (Figs. 1A-B and 2A). Upon consulting endocrinologists and nephrologists about the patient’s condition, it was concluded that the patient had metabolic bone disease-related hypophosphatasia osteomalacia. Besides, conservative treatment was chosen for the femoral neck fracture, which was later reviewed in 2017 to suggest healing of the femoral neck fracture (Figs. 1C and 2B). The patient had been treated symptomatically with salmon calcitonin, oestriol, and vitamin D during the period. However, he still suffered from chronic hypophosphatemic disease, in spite of the treatment. In 2018, the patient presented to our hospital with severe pain in the left thigh without any obvious causative factors. A pelvic X-ray suggested incomplete healing of bilateral femoral neck fractures and concomitant left intertrochanteric fracture of the femur (Fig. 1D). Subsequently, the patient was operated on in March 2018 for fracture incision and reduction with internal fixation placement. As the patient lay flat under general anesthesia, the surgeon made a lateral longitudinal incision of the femur about 15 cm long and incised the skin, subcutaneous tissue, fascia, and iliotibial bundle layer by layer, bluntly separated the muscles and incised the joint capsule. In addition to exposing the fracture end of the left femur under the rotor, peeling off the periosteum of the two severed ends, cleaning up the accumulated blood, and straightening the fracture end was done using the maneuver.

After the intraoperative X-ray was taken and the fracture end was ideally restored, a 7-hole locking plate was used, and 5 locking screws and 3 hollow nails were screwed into the plate sequentially for fixation. Intraoperative radiography showed that the position of the plate and the alignment of the fracture end were good. Besides, the function of the left hip joint could be improved. Finally, the wound was irrigated and sutured layer by layer. During the 2-year postoperative follow-up, imaging showed a secure internal fixation and good healing of the fracture (Fig. 1E-G). Subsequently, due to the COVID-19 epidemic, the patient was unable to return to the hospital for further follow-up after 2021. It is unknown how the patient recovered from his left femur fracture in 2022–2023. In March 2024, the patient presented to our hospital with a deformity of the appearance of the left thigh without any obvious triggers. The X-rays suggested bilateral proximal femur fractures, especially on the left side of the proximal femur. Comparing the previous X-rays, the patient’s 2018 subtrochanteric femur fracture had healed, with the loosening of the internal fixation device and a newly developed fracture of the left femoral stem (Fig. 1H). The cause of the patient’s re-fracture was unknown, as he had recurrent hip pain and prolonged bed rest after surgery in 2018, and he denied any history of trauma.

The patient denied a family history of bone illness and genetic problems linked to tumors, as well as underlying diseases related to diabetes, kidneys, and cardiovascular systems. At birth, he was healthy, growing and developing normally. Since 2015, however, he has been suffering from progressive rickets. None of the six siblings in the family had a history of similar fractures. The patient had one daughter, who was ordinaril fit and had a normal growth and development process.

**Fig. 3:**
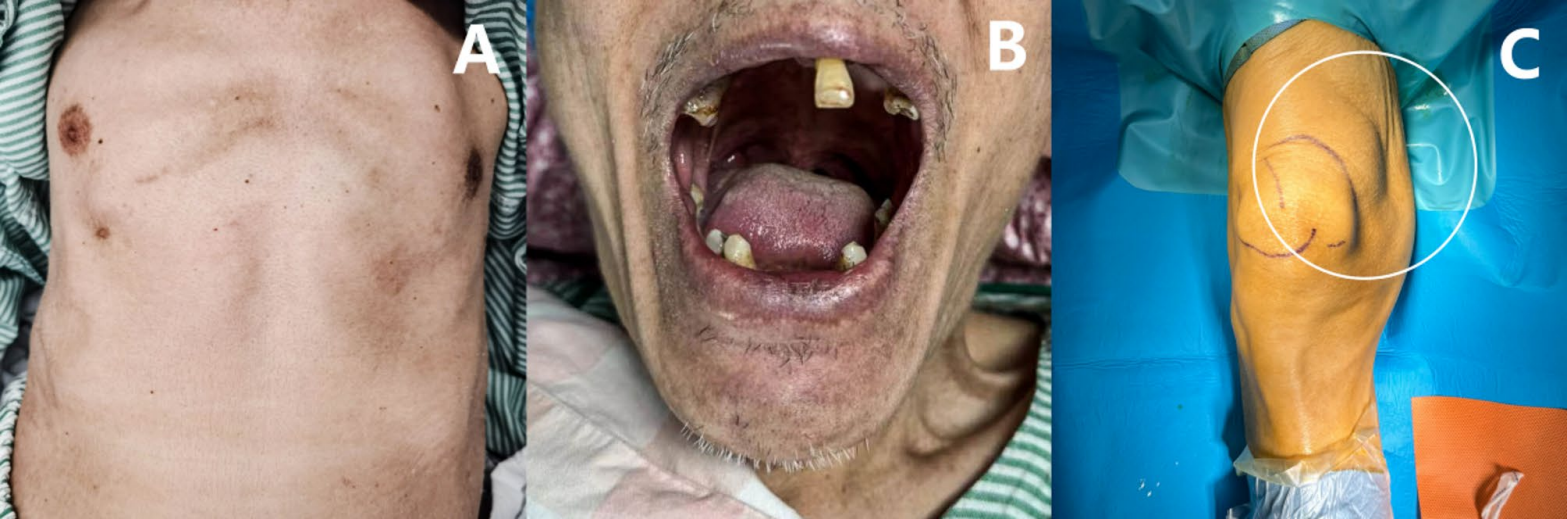
Images of the patient’s physical appearance. The patient’s chest was deformed, barrel-chested (**A**), and multiple teeth were seen to be missing (**B**). The patient had a palpable mass in the right suprapatellar bursa, which was hard, well-defined, and poorly mobile (**C**)

Upon physical examination, the patient revealed multiple missing teeth, dry, flaky skin over the body, rickets, barrel chest and no abnormalities on cardiac auscultation (Fig. 3). There was a palpable 4*3*2 cm mass on the patella of the right distal femur, with a distinct boundary, a firm texture, and limited movement. There was, however, no discomfort with the mass. There was normal blood flow, symmetrical and consistent sensation in both limbs, and a cast was applied to the left lower limb.

**Fig. 4:**
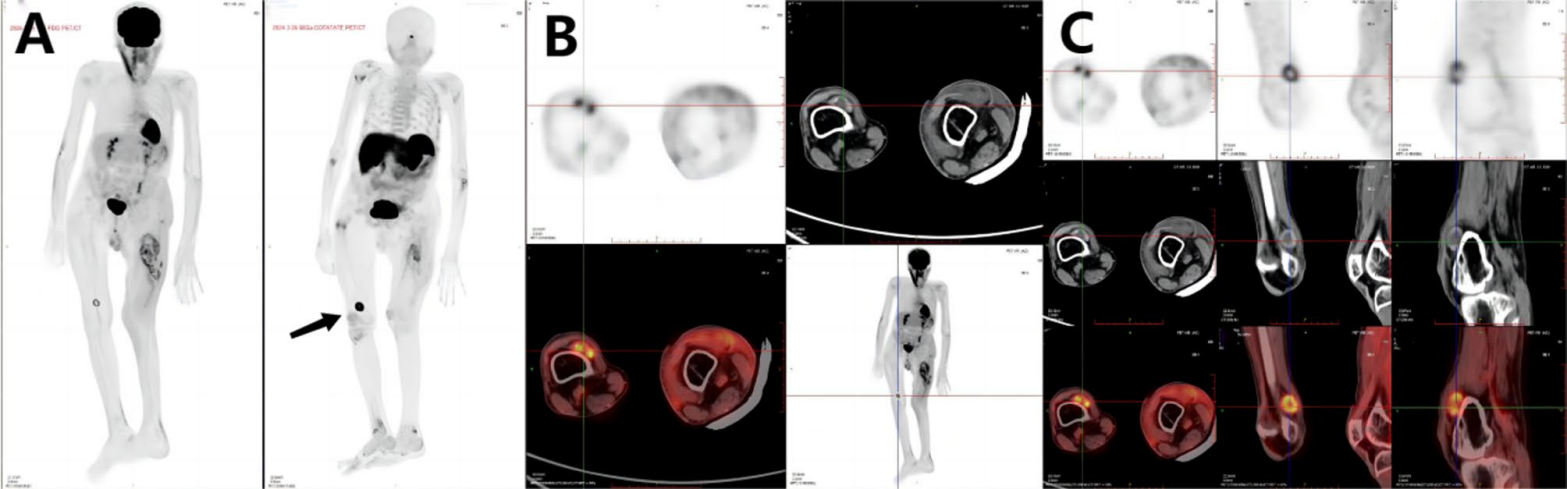
Complete body and tumor localization images from 68 Ga-DOTA-TATE PET/CT

By reviewing the patient’s relevant examinations from 2015 to 2024, human leucocyte antigen b27 (HLA-B27), antistreptolysin o (ASO), rheumatoid factor (RF) and tumor markers were not abnormal. The rest of the laboratory findings suggested that the patient was chronically vitamin D deficient and hypophosphatemic (Table 1). The patient had osteoporosis, according to results from years of monitoring bone densitometry (Table 2). We significantly suspected TIO based on the patient’s medical history and lab results; hence, a full 68 Ga-DOTA-TATE PET/CT scan was performed on the patient. PET/CT results suggested: the right case of suprapatellar bursa cystic solid nodule; FDG PET showed a mild increase in the bursa wall’s metabolism; and DOTATATE PET/CT suggested a significant increase in metabolism. In summary, the patient was considered for a possible diagnosis of PMT (Fig. 4).

**Table 1:**
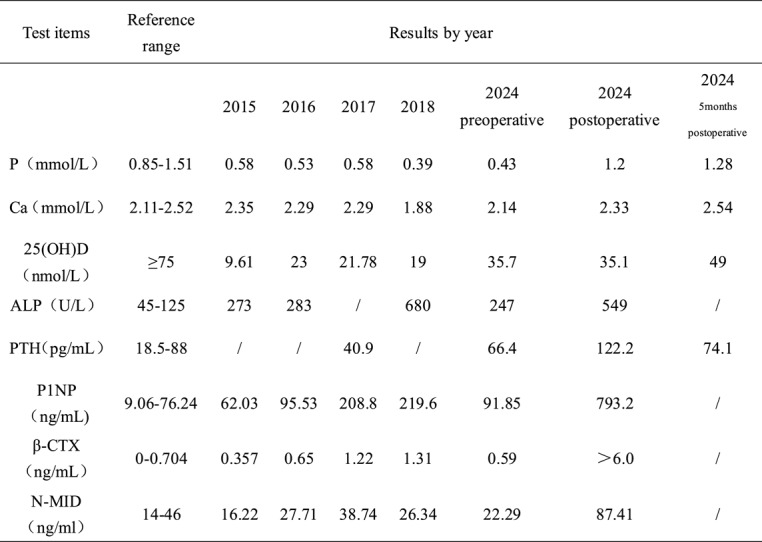
Bone metabolism−related biochemical parameters during 2015–2024

**Fig. 5:**
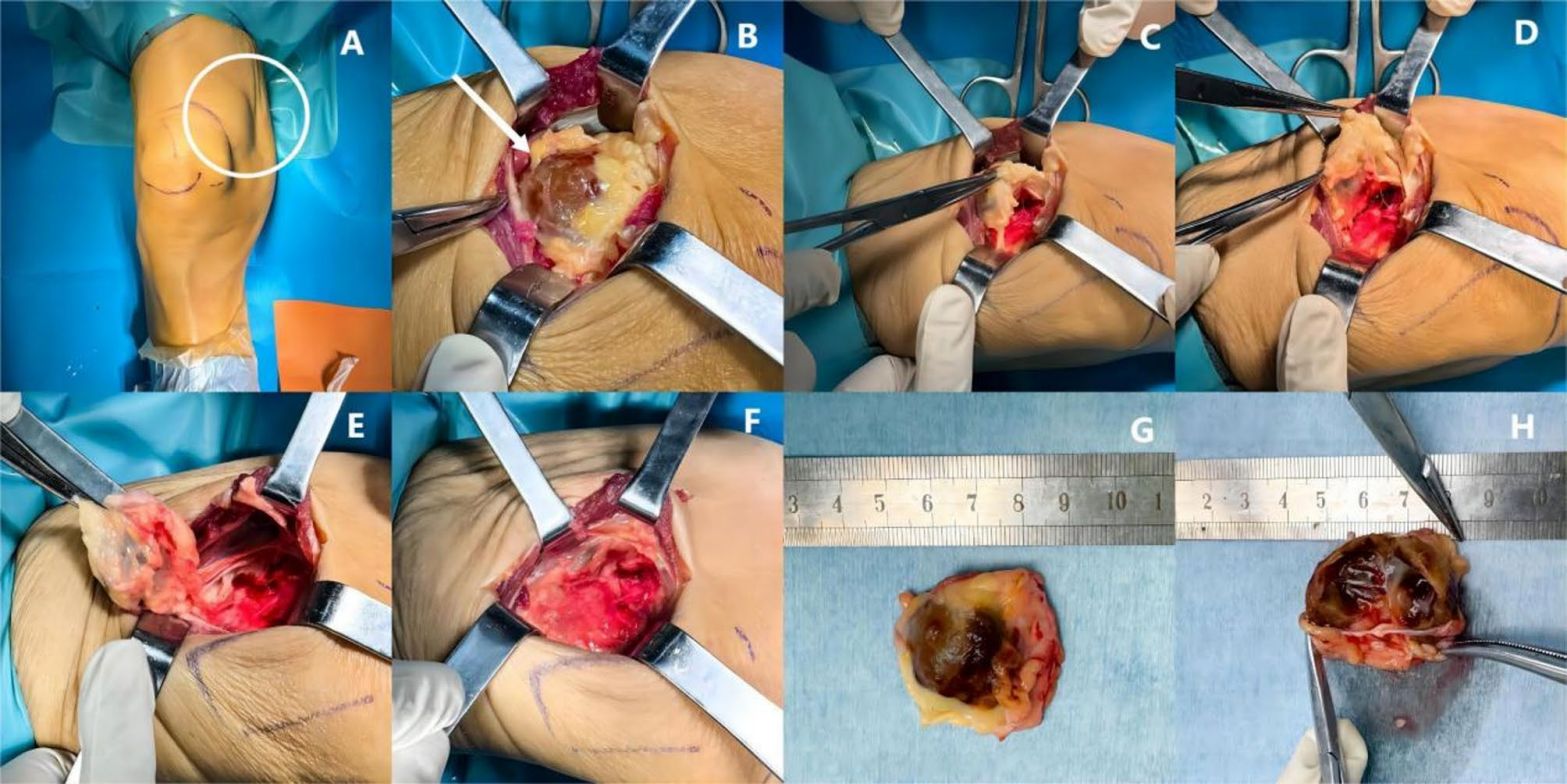
Intraoperative conditions. **A** The mass was palpated. The lesion was noted as being located at the right suprapatellar bursa based on the preoperative PET-CT results. **B** Cut layer by layer through the skin to reveal the medial femoral muscle and subcutaneous tissue, and then follow the muscle’s outline to the tumor. **C**, **D**, **E** Total dismantling of the mass and a rough division of the mass along the edges. **E** The mass was entirely removed. The wound was irrigated. **G**, **H** After the mass was entirely separated, a brownish-brown fluid spilled out of it. The mass measured approximately 2.7 * 2.5 * 1.4 cm and had a smooth surface, a dark red color, and a firm texture

**Fig. 6:**
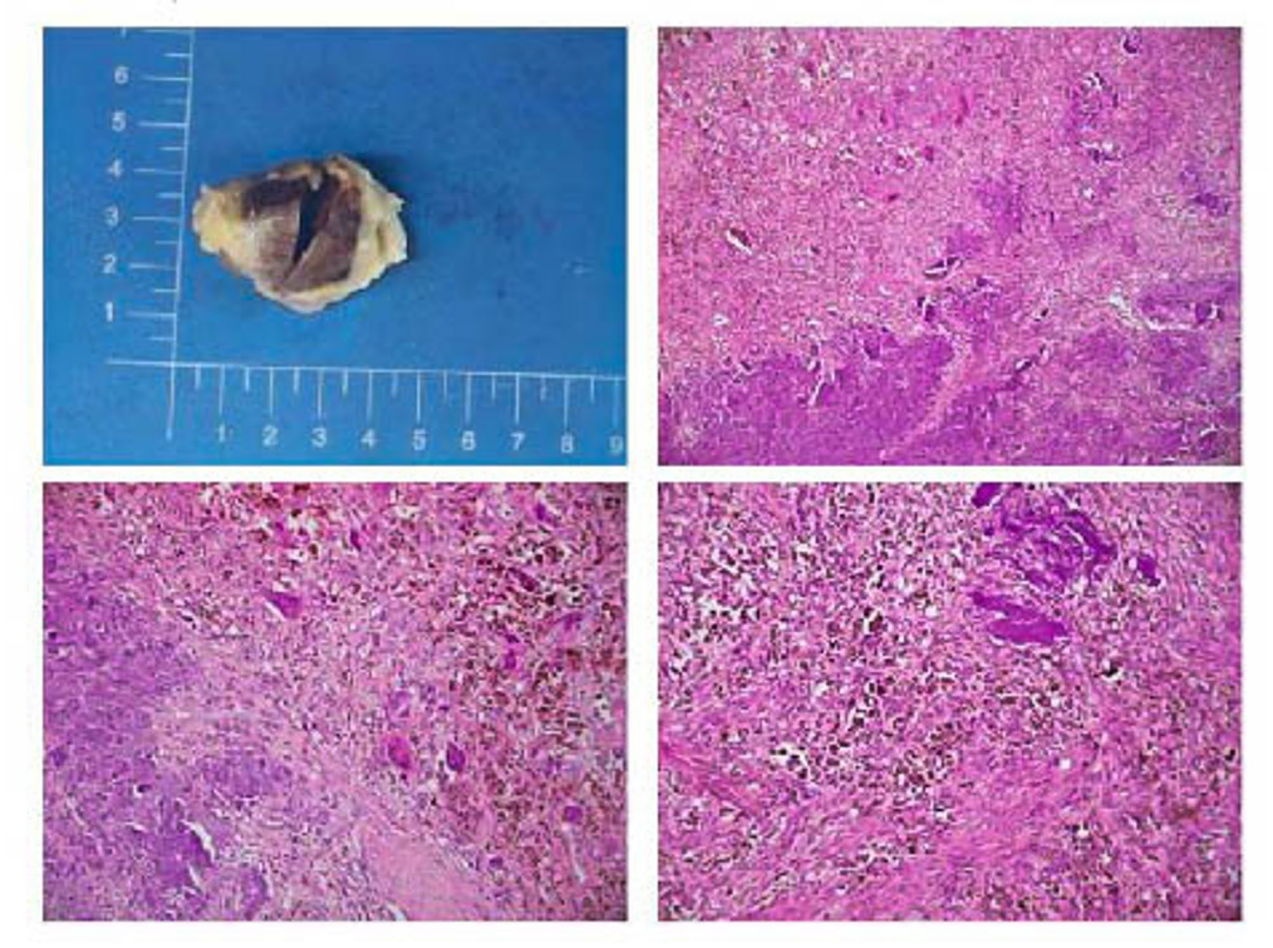
Pathological findings of the tumor. Under a microscope, the tumor cells were sparsely, densely, and diffusely distributed. They were also short, spindle-shaped, or stellate, with partially powder-stained cytoplasm and a small percentage of light-stained and vacuolated cells. The capillaries were numerous, and the background displayed a significant number of irregularly granular or cotton-wool-like calcifications and vitreous lesions, along with an important amount of ferritin deposition, as well as scattered inflammatory cells and infiltration of multinucleated giant cells

Therefore, the right suprapatellar bursa lump was excised. The left upper femur fracture was reduced and internally fixed (Fig. 5). Postoperative follow-up X-rays revealed that the fracture was well-displaced (Fig. 1I). Pathologic findings of the mass specimen were consistent with the PMT (Fig. 6). The patient was ultimately diagnosed with PMT based on imaging and pathologic findings, which was verified by the gradual rebound of blood phosphorus postoperatively compared with before.

**Fig. 7:**
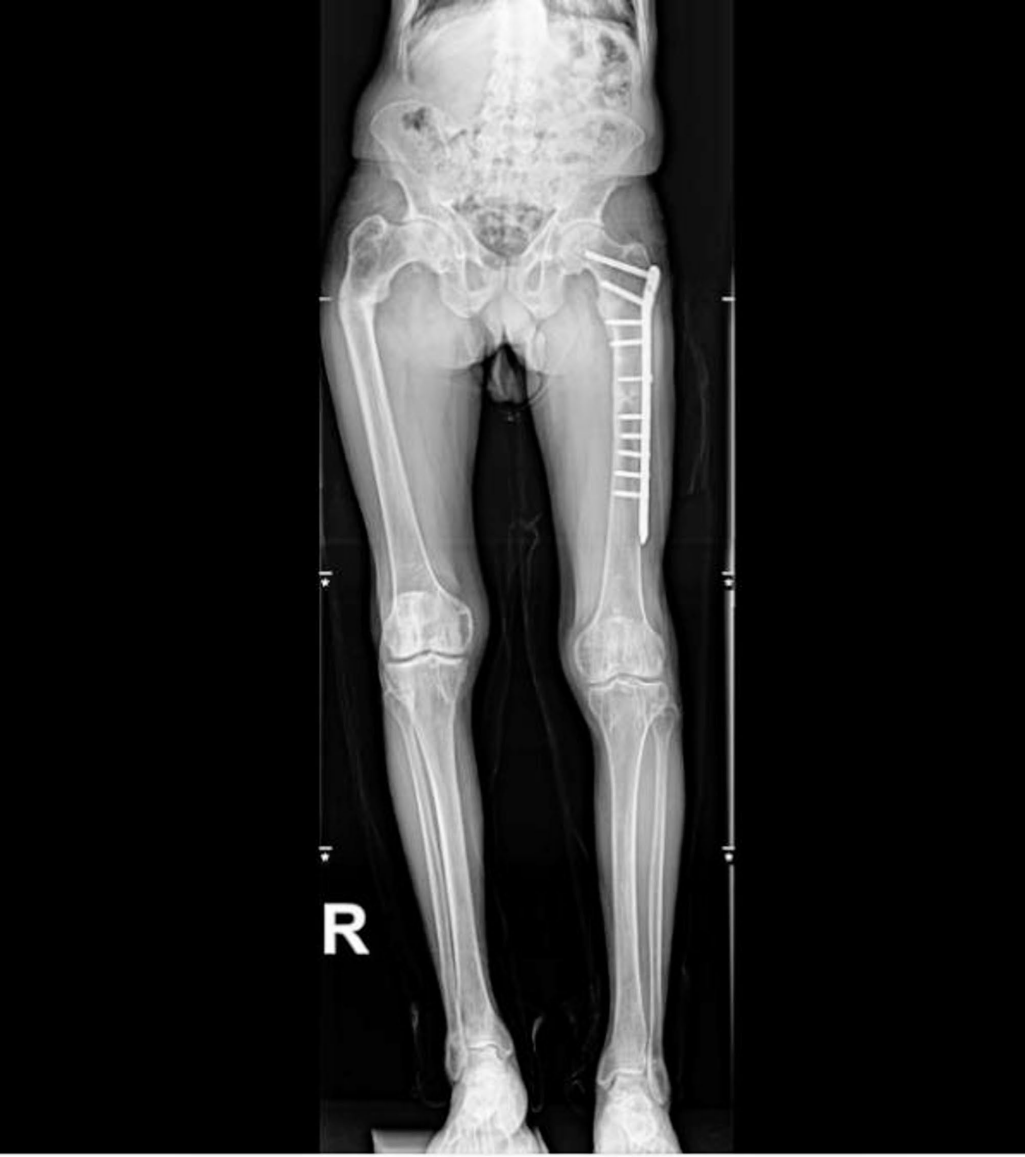
A follow-up examination more than 1 year after the operation showed good healing of the fracture end on April 27, 2025

The patient was followed up for 1 year after surgery. On April 27, 2025, the patient returned to the outpatient clinic for review, complaining of no further symptoms of muscle twitching in the lower extremities and hip pain. As prescribed, he began walking on the ground using a walker. Due to his unequal walking gait limp of both lower limbs, he had slight pain and discomfort at the left fracture site when walking. The test results showed that the blood phosphorus was 0.97mmol/L and the blood calcium was 2.54mmol/L. The full-length DR of both lower limbs showed that the fracture end of the right femur was healing well (Fig. 7).

## Discussion

PMT is a rare tumor that occurs in soft tissue or bone. In previous reports, PMT has been described to be mostly benign, with only a minimal few malignant cases reported [2]. The concept of PMT was initially proposed by Weidner and Santa in 1987. Later on, the majority of PMT cases have been clarified to be classifiable as a phosphaturic mesenchymal tumor mixed connective tissue variant (PMTMCT) [4].

PMT is currently considered to be a major etiological cause of TIO [5]. A retrospective study in China collected data on 144 patients with PMT. The interval between onset and definitive diagnosis was 2.9 years, and the interval between onset and tumor resection was 5.4 years, with a 95.1% misdiagnosis rate [6]. The patient in this case experienced 9 years from the onset of symptoms to definitive diagnosis, during which he was diagnosed with metabolic bone disease, primary bilateral hip arthropathy, and hypophosphatasic osteomalacia. He had also perfected PET-CT to rule out a tumor, but unfortunately, no tumor foci were detected.

### Bone turnover markers (BTMs)

**Fig. 8:**
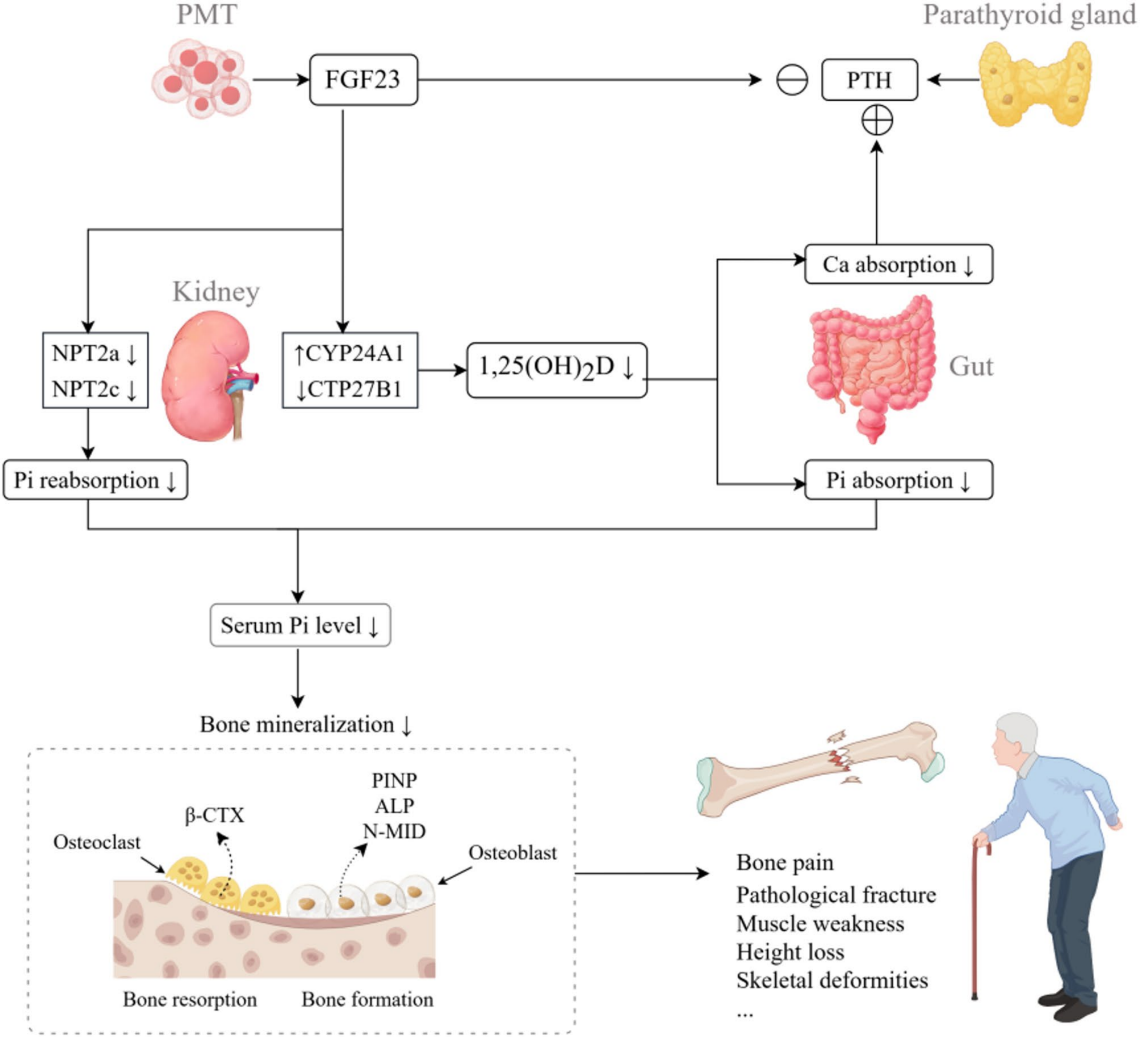
Pathogenesis of tumor-induced osteomalacia. (By Figdraw). PMT expresses a large amount of FGF23, which acts on the kidney to reduce the expression of NPT2a and NPT2c, thereby reducing phosphorus reabsorption. It also inhibits renal 1,25(OH)_2_D production by decreasing CTP27B1 expression and promotes 1,25(OH)_2_D degradation by increasing CYP24A1 expression, indirectly leading to decreased intestinal absorption of calcium and phosphorus. In addition, FGF23 can directly inhibit PTH secretion. However, due to the positive stimulation of the parathyroid glands by low blood calcium, PTH can be maintained at a relatively normal level. The above hormonal effects together result in low blood phosphorus and high urinary phosphorus. Low blood phosphorus leads to decreased bone mineralization and eventually to osteomalacia, which manifests as bone pain, pathological fractures, muscle weakness, rickets, and skeletal deformities

It is well known that the main cause of osteochondrosis in PMT is the massive secretion of a phosphoregulatory factor FGF23 by the tumor tissue [2]. Under normal conditions, FGF23 is mainly produced by osteoblasts and osteoclasts. It coordinates and restrains with parathyroid hormone (PTH), 1,25(OH)_2_D through three target organs, namely, the small intestine, bone, and kidneys, which work together to maintain the blood phosphorus concentration at a relatively constant level. When the concentration of FGF23 in the blood is abnormally increased due to the secretion of PMT, the blood phosphorus in the body is decreased [7]. Blood phosphorus decrease will cause bone matrix mineralization anomalies and lead to osteomalacia [8]. At the same time, in order to combat the occurrence of osteomalacia, alkaline phosphatase (ALP) will rise compensatorily to promote bone mineralization [9, 10] (Fig. 8). Based on the above physiopathological process of PMT, patients with PMT tend to have decreased blood phosphorus, increased urinary phosphorus and ALP, and normal or decreased blood calcium, 25(OH)D and parathyroid hormone (PTH). A few numbers of patients also develop secondary hyperparathyroidism, which in turn leads to elevated PTH levels [11, 12].

Reviewing the summary of the patient’s laboratory findings (Table 1), we can see that before tumor resection the patient’s blood phosphorus level was significantly lower and ALP was heightened. In addition, blood calcium, 25(OH)D, PTH, β-C-terminal telopeptide of type 1 collagen (β-CTX), N-terminal mid-fragment of osteocalcin (N-MID), and procollagen type I N-terminal propeptide (PINP) were basically at normal levels, which was in line with the results of previous related studies [13, 14]. Unlike the ALP, the specific indicators β-CTX, N-MID, and PINP, which respond to bone resorption and formation, were not significantly elevated during the years of the onset of the disease. However, after tumor resection, β-CTX, N-MID, and PINP appeared to be substantially elevated, and blood phosphorus returned to normal levels, suggesting that the tumor significantly improved the environment for PMT bone metabolism. Previously, although there are articles pointing out that β-CTX, N-MID, and PINP in PMT patients will be at normal levels, there has not been a study of long-term observation of BTMs in patients with PMT [13, 14]. In addition, the clinical diagnostic significance of BTMs for PMT needs to be further explored.

### Radiographic

X-ray radiographs are the first choice for imaging hip fractures in the elderly, which can assess the overall skeletal changes caused by TIO as a whole. Mild cases may present only with decreased bone density, while severe cases may present with fractures [14]. Bone densitometry can indicate a significant decrease in bone mineral density. The diagnostic criteria for osteoporosis can be met in most patients [15]. Epidemiological surveys have shown that the prevalence of osteoporosis in China is as high as 32% (10.7% in males and 51.6% in females). Because bone dual-energy X-ray absorptiometry (DXA) scans usually show a decrease in bone mineral density (BMD) due to a decrease in bone mineralization. Patients with TIO are often misdiagnosed as osteoporosis in the clinic and are treated only with supplementation of calcium and vitamin D and other anti-osteoporosis medications. Thus, the time required to diagnose TIO in patients is greatly prolonged [6, 16]. According to this patient’s previous BMD results over several years (Table 2), osteoporosis may have been present for a long period of time. Metabolic bone disease, osteoporosis, and pathological fractures had been diagnosed during this period, with the exact etiology unknown and anti-osteoporotic treatment ineffective, thus prolonging the time to diagnosis.

**Table 2:** Bone densitometry parameters during 2015–2024

Bone density data	Results by year					
	2015	2016	2017	2018	2021	2024
lumbar vertebra BMD (g/cm²)	0.891	0.866	0.809	0.788	0.876	0.76
lumbar vertebra T-Score	−1.8	−2.0	−2.6	−2.8	−2.0	−3.0
left femur BMD (g/cm²)	0.443	0.506	0.466	0.454	0.532	0.627
left femur T-Score	−3.6	−3.1	−3.4	−3.5	−3.3	−2.7

Furthermore, the localization of the primary tumor by PET/CT is necessary due to the variable growth location and often the small size of PMT [17]. 18 F-FDG, as the most commonly used tracer in PET/CT, has good diagnostic value for tumor tissues with increased glucose metabolism. Thus, it is widely used in lung tumors, brain cancers, gastrointestinal tract tumors, metastatic hepatocellular carcinoma, pancreatic cancers, breast cancers, lymphoma, melanoma, and other tumors. However, the relatively poor sensitivity of 18 F-FDG PET/CT for PMT may be mainly due to the benign nature and low metabolic activity associated with PMT [18]. In this case, when the patient underwent 18 F-FDG PET/CT in 2018, it was only suggestive of generalized multiple fractures and extensive osteoporosis, with no definite abnormal hypermetabolic lesions. It has been shown that PMT expresses growth inhibitory receptors, which can be visualized using growth inhibitor analogs on single photon emission CT or positron emission CT. Besides, DOTA-TATE binds specifically to tumor foci expressing somatostatin receptors. What’s more, it has also been shown that 68Ga-DOTA-TATE PET/CT is the most effective in localizing PMT and reducing diagnostic time [19]. When he underwent 18 F-FDG and 68Ga-DOTA-TATE PET/CT again on 2024-3-22, he was found to have a right suprapatellar cystic solid nodule measuring approximately 2.6cm*1.3cm*2.5 cm, with mild metabolic increase of the cystic wall on FDG PET/CT (SUVmax:3.0) and significant metabolic increase on DOTA-TATE PET/CT (SUVmax:14.8). SUVmax:14.8). Through years of tortuous diagnosis and treatment of this case, it was confirmed that 68Ga-DOTA-TATE is indeed more advantageous than 18 F-FDG in the diagnosis of PMT.

### Medication

The established treatment for TIO is complete surgical resection of the tumor [20]. However, tumors are usually small and occur in soft tissue or bone, making them difficult to locate and delaying diagnosis and treatment. 35 to 40% of tumors cannot be localized [21, 22]. When tumors cannot be localized or resected, medication is often needed to improve symptoms and reduce skeletal complications. The treatment consists of multiple daily oral doses of phosphate and active vitamin D [23]. However, this supplemental therapy has had limited therapeutic efficacy in the treatment of this type of skeletal deformity associated with it. Burosumab is a fully human monoclonal antibody that binds and inhibits the activity of excess circulating FGF 23, restoring phosphate homeostasis. It is the only therapy approved by the U.S. Food and Drug Administration (FDA) for the treatment of TIO patients with unresectable or unlocatable tumors. Based on available clinical research data, the FDA has approved burosumab for patients with TIO with doses ranging from 0.5 mg/kg every 4 weeks up to 2.0 mg/kg every 2 weeks [24].

Burosumab was reported to be generally well tolerated in clinical trials in both pediatric and adult populations. Discontinuations and deaths related to treatment-emergent adverse events (TEAE) have not been reported in large pediatric and adult clinical trials [25]. However, most of the current published evidence on TIO is in the form of case studies or case series [26]. In the absence of reliable data, further data are needed to standardize appropriate dosage regimens and to improve the observation of their long-term efficacy and safety. Burosumab may be a promising pharmacological treatment for TIO that can be used in situations where definitive surgery is not indicated or where no tumor is detected.

### Surgical program

As there have been no reports of internal fixation device failure after fracture surgery in PMT patients, the causes of their internal fixation failure were investigated. The patient suffered a left femoral subrotator fracture with no apparent causation back in 2018. In people with poor bone quality, low-energy injuries can also fracture this area [27]. For these types of fractures, there is consensus for internal fixation placement surgery if there are no absolute contraindications to surgery. In addition to accelerating the patient’s rehabilitation by allowing him to perform functional exercises on the ground, this technique improves his quality of life and helps him regain hip function more rapidly. For subtrochanteric fracture, the preferred treatment is reduction and internal fixation. Excellent reduction of the fracture is a prerequisite for internal fixation. There are two types of internal fixation: (1) extramedullary fixation system, represented by dynamic hip screw; (2) intramedullary fixation system, represented by proximal femoral nail anti-rotation (PFNA) [28]. After evaluation, the attending surgeon chose to use a proximal femoral locking compression plate (PFLCP) for fixation, which is a new type of internal fixation device in the last decade or so. It is suitable for inter- and sub-trochanteric fractures of the femur and is characterized by the fact that there are compression and locking holes on the plate. It can be used as a bridging plate as well as for compression of the fracture block to make the fracture more stable, which can achieve good results [29–31].

In 2018, our patient was selected for internal fixation with PELCP after experiencing a subtrochanteric fracture which healed well on several postoperative reviews. In March 2024, the patient unexpectedly suffered an ipsilateral femoral stem fracture without any significant external force. An X-ray revealed a femoral stem stress fracture at the end of the main nail, suggesting failure of the internal fixation device. It has been reported in the literature that poor integrity of the lateral wall of the femur, small thickness of the lateral wall, more osteoporosis, functional fracture reduction, unstable fracture, comorbid medical disorders, and a greater than 25-mm cusp distance are all risk factors for internal fixation failure in intertrochanteric femur fractures [32–34]. A related Meta-analysis stated that the rate of internal fixation failure in intertrochanteric and subtrochanteric fractures treated with PFLCP was 20.05%. The postoperative internal fixation failure rate was higher in those with age > 60 years and incomplete posterior medial wall of the rotor region. In contrast, there was no significant relationship between fracture site, time to first postoperative partial weight bearing on the floor, and PFLCP fixation failure [35]. In addition to this, the incidence of internal fixation failure was much higher in unstable fractures than in stable fractures, and the incidence of internal fixation failure was much higher in nail plate systems than in intramedullary systems [32].

Recall that in this case, the patient had bilateral femoral neck fractures in 2015. Following conservative treatment, most of the bilateral femoral neck fractures were seen to have healed in 2017. In 2018, the patient presented with a left subtrochanteric fracture of the femur, which was subsequently successfully operated on, with postoperative imaging suggesting good healing of the fracture. We thus concluded that the stabilizing effect of PELCP for this patient was considerable. In terms of the reason why the patient suffered bilateral femoral stem fractures in 2024, we consider that the PELCP plate was not responsible for the failure of internal fixation. Due to the long-term presence of hypophosphatemia, osteomalacia, and osteoporosis in PMT patients, bone quality is greatly reduced. As long as the primary lesion is not eradicated, fractures will occur repeatedly in all bones throughout the body, and resection of the primary tumor lesion is the key to PMT’s treatment. It is worth mentioning that perioperative management is also important. In this case, despite poor nutritional status and weak muscle strength, the patient recovered well with postoperative albumin supplementation, as well as a calcium and protein-rich diet. Patients should be weight-bearing on the floor for a longer period of time after surgery than the general population and follow-up visits should be relatively frequent, with a dynamic review of biochemical indexes. Besides, anti-osteoporosis treatment should be standardized, with the use of bisphosphonates, teriparatide, desutumumab, and other drugs, if necessary.

## Conclusion

In summary, this study brings attention to an often-overlooked disease in the clinical setting of periprosthetic fracture with hardware failure. In clinical practice, if a patient is found to have multiple fractures throughout the body, bone metabolism related tests indicative of chronic hypophosphatemia and osteoporosis, and fails to respond to medications, physicians should use 68 Ga-DOTA-TATE to detect PMT. In cases of hypophosphatemia with musculoskeletal symptoms of undetermined origin, burosumab can be considered as a treatment option.

## Data Availability

The datasets used and analysed during the current study are available from the corresponding author on reasonable request.

## Abbreviations

TIO: Tumor-induced osteomalacia
FGF23: Fibroblast growth factor 23
PMT: Phosphaturic mesenchymal tumor
BTMs: Bone turnover markers
PET/CT: Positron Emission Tomography/ Computed Tomography
HLA-B27: Human leucocyte antigen b27
ASO: Antistreptolysin o
RF: Rheumatoid factor
1,25(OH)_2_D: 1,25 dihydroxy vitamin D
PMTMCT: Phosphaturic mesenchymal tumor mixed connective tissue variant
ALP: Alkaline phosphatase
B-ALP: Bone-alkaline phosphatase
PPi: Pyrophosphate; Pi: Phosphate
PINP: Procollagen type I N-terminal propeptide
β-CTX: β-C-terminal telopeptide of type 1 collagen
N- MID: N−terminal mid−fragment of osteocalcin
PFLCP: Proximal femoral locking compression plate
PFNA: Proximal femoral nail anti-rotation
TEAE: Treatment-emergent adverse events
